# Eating Behaviors and Dietary Patterns of Women during Pregnancy: Optimizing the Universal ‘Teachable Moment’

**DOI:** 10.3390/nu13093298

**Published:** 2021-09-21

**Authors:** Maryam Kebbe, Emily W. Flanagan, Joshua R. Sparks, Leanne M. Redman

**Affiliations:** Reproductive Endocrinology and Women’s Health Laboratory, Pennington Biomedical Research Center, Baton Rouge, LA 70808, USA; maryam.kebbe@pbrc.edu (M.K.); emily.flanagan@pbrc.edu (E.W.F.); joshua.sparks@pbrc.edu (J.R.S.)

**Keywords:** cross-sectional, diet, nutritional status, obesity, pregnancy

## Abstract

Understanding women’s perceptions of eating behaviors and dietary patterns can inform the ‘teachable moment’ model of pregnancy. Our objectives were to describe eating behaviors and dietary patterns in pregnancy. This was a cross-sectional, national electronic survey. Women were ≥18 years of age, living in the United States, currently pregnant or less than two years postpartum, and had internet access. Age, education, race, and marriage were included as covariates in ordinal and binary logistic regressions (significance *p* < 0.05). Women (*n* = 587 eligible) made positive or negative changes to their diets, while others maintained pre-existing eating behaviors. The majority of women did not try (84.9 to 95.1% across diets) and were unwilling to try (66.6 to 81%) specific dietary patterns during pregnancy. Concerns included not eating a balanced diet (60.1 to 65.9%), difficulty in implementation without family (63.2 to 64.8%), and expense (58.7 to 60.1%). Helpful strategies included being provided all meals and snacks (88.1 to 90.6%) and periodic consultations with a dietitian or nutritionist (85 to 86.7%). Responses differed across subgroups of parity, body mass index, and trimester, notably in women with obesity who reported healthier changes to their diet (*p* < 0.05). Our study underscores the importance of tailoring care early to individual needs, characteristics, and circumstances.

## 1. Introduction

Ensuring proper nutrition is a cornerstone for prenatal care. Nutritional status during pregnancy not only influences the woman’s health, but also pregnancy outcomes and fetal health and development. Pregnancy is accompanied by many physiological changes that alter both energy and nutrient demands. Pregnant women in turn require different energy and nutritional needs from the pre-pregnancy period. As per recommendations from the Institute of Medicine (IOM), daily recommendations for most vitamins and minerals increase and certain micronutrients, such as folate, are deemed essential for optimal fetal development [1,2]. While no specific dietary pattern is recommended, a variety of foods should be consumed to meet the nutrient and energy demands of pregnancy in accordance with the IOM guidelines and the 2020–2025 Dietary Guidelines for Americans [2,3]. That is, a woman’s pre-pregnancy body mass index (BMI) informs the total amount of weight gain and rate of weight gain during pregnancy, both of which are important to achieve optimal maternal and neonatal health outcomes [2,4]. 

Pregnancy is often perceived as a teachable moment for lifestyle behavior change due to increased motivation for a healthy pregnancy and more frequent contact with health care professionals [5]. A large proportion of pregnant women consider following an appropriate diet as one of their most important learning needs during pregnancy [6]. Yet, pregnant women may not be meeting requirements stipulated in dietary guidelines and/or nutritional recommendations [7,8]. As a result, many women present with nutrient inadequacies during their pregnancy [9,10,11,12]. 

Suboptimal dietary practices may arise from complexities in navigating the dietary landscape that is often overwhelming, ambiguous, and conflicting [13,14,15,16,17]. In addition, pregnancy may be perceived by some as a time to succumb to cravings and to pause rigid rules around diet or to disproportionately increase calories to ‘eat for two’ [18]. Multifaceted factors such as sociodemographic diversity, anthropometrics, individual characteristics, and interpersonal relations can further influence dietary behaviors of pregnant women [14,19]. For example, a lower diet quality has been observed in women with overweight and obesity compared to normal-weight women in the first trimester and in less educated, younger women who reside in an urban setting in late pregnancy [20]. A woman’s individual characteristics and circumstances may dictate the conditions necessary to create a teachable moment. Therefore, lessons that predict or promote dietary behavior change during pregnancy may not need to be universal, but personalized instead.

In order to capitalize on the teachable moment model, it is important to understand women’s perceptions of eating behaviors and dietary patterns during pregnancy, and how they feel about specific dietary patterns and practices. While previous research described changes to women’s consumption of specific foods during pregnancy [21,22], none has examined perceptions towards specific eating behaviors and modern dietary patterns. The overall objective of this study was to describe eating behaviors and dietary patterns in pregnancy. Specifically, this study sought to examine perceived changes to eating behaviors, as well as attempts, willingness, safety, concerns, and helpful strategies in relation to adopting specific dietary patterns. As dietary patterns might be influenced by past pregnancy experience, weight status, or pregnancy adaptations resulting in nausea and appetite changes, we also studied these outcomes in relation to parity, BMI, and by trimester. 

## 2. Materials and Methods

### 2.1. Study Design

This was a cross-sectional national, electronic survey that targeted pregnant or recently pregnant women between December 2020 and March 2021. The Modern Mommy Bites survey aimed to capture perceptions of modern dietary and physical activity habits during pregnancy, the latter being outside the scope of this paper. Although the survey did not use validated questions, it was developed, piloted, and refined by research members of the Reproductive Endocrinology and Women’s Health Laboratory at the Pennington Biomedical Research Center. The survey included a short form (*n* = 35 questions) and a long form (short form plus five additional questions and an opportunity for free-text responses). The short form consisted of four domains: (i) participant demographics and self-reported anthropometrics (e.g., height, pre-pregnancy weight), (ii) current eating and exercise behaviors during pregnancy, (iii) perceptions towards modern eating behaviors and patterns during pregnancy, and (iv) perceptions towards exercising during pregnancy. The survey began with the short form questions and if they were willing to continue, participants were asked to answer additional questions around concerns and safety of following select dietary patterns during pregnancy. They were also provided with an open-ended text box to share any additional details regarding their thoughts on dietary and physical activity habits during pregnancy. Specific dietary patterns were chosen given their popularity amongst the public community, and given the lack of investigation by the research community with respect to the pregnant population. Understanding women’s perceptions of these dietary patterns during pregnancy will inform future interventions that are personalized, relevant, and effective for pregnant women.

### 2.2. Recruitment

To be eligible for inclusion, participants were required to be women, ≥18 years of age, living in the United States, currently pregnant or less than two years postpartum, and to have internet access. The electronic survey, hosted on Research Electronic Data Capture (REDCap^®^), an online, secure sharing platform [23], was disseminated via paid, targeted advertisements through social media platforms (Facebook, Twitter, and Instagram), the PBRC website and email listserv, and word of mouth. Advertisements stated the purpose of the study and the opportunity to enter a drawing for one of ten $50 gift cards (Clincard, GreenPhire). 

### 2.3. Data Collection

Before providing consent and completing the survey, participants were informed of the purpose of the study, expected time for completion (~15 min for the short form and ~20 min for the long form), and expectations for answering survey questions. Responses were anonymous and de-identified for all participants. 

The primary survey questions used to investigate our outcomes were:

1. Compared to before you were pregnant, how would you rate your changes to the following eating behaviors when pregnant? (If you are currently not pregnant, please answer for when you were pregnant). Available responses were: A lot less, A little less, About the same, A little more, A lot more, and I don’t know. Eating behaviors included taking a daily multivitamin, healthfulness of food, size of meals, amount of food, sugar, fat, salty foods, fried or fast foods fruit, vegetables, and fiber, time between last meal/snack and bed time, night-time eating/drinking, and snacking, breakfast, sugar-sweetened beverage, and calorie-counting frequency.
2. When pregnant, have you followed any of these diets? If not, would you be willing to work with a dietitian to follow one of these eating patterns during the second or third trimester of pregnancy? Available responses were: I have tried this diet during pregnancy; I have tried this diet during pregnancy and did not like it; I have not tried this diet and would not be willing to try it during my pregnancy; and I have not tried this diet but would be willing to try it during my pregnancy. Dietary patterns included Paleo, Ketogenic, Atkins, or similar low-carb diet, Mediterranean or DASH diet, Whole-30 diet, Vegetarian diet, and Vegan diet, calorie-counting.
3. How safe would you rate following these diets during your second and third trimester of pregnancy? Available responses were: Not at all safe; Not very safe; Somewhat safe; Very safe; and I don’t know. Dietary patterns included Paleo, Ketogenic, Atkins, or similar low-carb diet, Mediterranean or DASH diet, Whole-30 diet, Vegetarian diet, Vegan diet, calorie-counting, low-fat foods, low-carb foods, and low- or no sugar foods.
4. What concerns would you have about following these eating plans during your second or third trimester of pregnancy? Available responses were: Concern for Vegetarian/Plant-based diet; Concern for Paleo Ketogenic, Atkins, or similar low-carb diet; Concern for both of these diets; and Not a concern for either of these diets. Concerns included not enjoying the food, being too hungry, getting nauseous, not eating enough food, not eating a balanced diet, being unsafe for woman’s health, being unsafe for the baby’s health, being unsuitable with busy schedule, being expensive, being hard to follow without a family.
5. Select if these things would make following each diet more helpful. Available responses were: Helpful for Vegetarian/Plant-based diet, Helpful for Paleo, Ketogenic, Atkins, or similar low-carb diet, Helpful for both of these diets, and Not helpful for either of these diets. Strategies included being provided protein shakes or bars to supplement own diet, being provided all meals and snacks, following the diet on only five days of the week, meeting with a dietitian or nutritionist periodically, and more frequent check-ins with doctors to ensure the baby is healthy.
6. Do you have any more thoughts or concerns on exercise or diet programs during pregnancy? Response was an open-ended text box.

### 2.4. Data Analysis

Data were exported from REDCap^®^ into SPSS v. 26.0 for analyses. Descriptive analyses (e.g., frequencies) were generated for sociodemographics and sub-domains of eating behaviors and patterns, namely previous attempts, willingness, safety, concerns, and helpful strategies. BMI was computed as height (m)/weight (kg)^2^ based on self-reported current weight if not currently pregnant or pre-pregnancy weight if currently pregnant. Self-reported pre-pregnancy weight is widely used in population studies and has been shown to be a reasonable estimate of weight at conception [24]. Weight status was classified as normal weight (18.5 ≤ BMI < 25 kg/m^2^), overweight (25 ≤ BMI < 30 kg/m^2^), and obesity (≥30 kg/m^2^). Outcome variables were categorized as three-level (less/maintain/more) or two-level (tried/not tried, willing/not willing, safe/not safe, concern/not a concern, and helpful/not helpful) responses. Chi-squared analyses were performed for each item within each sub-domain; to remain conservative, items that were *p* ≤ 0.2 were carried forward for regression analyses. Ordinal and binary logistic regressions were then computed for response items with three or two levels, respectively. As a next step, three-level responses (less vs. maintain vs. more) that were *p* ≤ 0.2 in our ordinal logistic regression model were categorized into three two-level responses (less vs. more, less vs. maintain, more vs. maintain) and were fitted into a binary logistic regression. Using three independent adjusted models, we examined whether responses differed by pregnancy characteristics, including parity (multiparous as a reference), weight status (normal weight as a reference), and trimester (third trimester as a reference). Age (≤30 years or >30), education (with a graduate degree or with less than a graduate degree), race (Caucasian or non-Caucasian), and marriage (married or non-married) were coded as two-level responses and included as covariates. Results are presented as odds ratios (95% confidence intervals (CI)) and significance at *p* < 0.05. Responses to the free-text box were manually coded using manifest, inductive content analysis by three independent researchers. Responses were read and re-read for familiarization, after which a coding scheme was developed, refined, applied to the entire dataset, and sorted into categories. Exemplar quotes of women’s eating behaviors and patterns during pregnancy were selected.

## 3. Results

### 3.1. Respondent Demographics

Overall, 671 women provided consent and initiated the survey. Out of 587 women who were eligible, the majority were older than 30 years of age (*n* = 318, 54.2%), Caucasian (*n* = 487, 83.2%), married (*n* = 485; 82.6%), multiparous (*n* = 434; 73.9%), had less than a graduate degree (*n* = 382; 65.1%), and had obesity (*n* = 175, 29.8%). Out of 394 women who were currently pregnant, the majority were in their second trimester of pregnancy (*n* = 181, 45.9%). The number of women varied by question and item, since dropouts occurred as the survey progressed.

### 3.2. Eating Behaviors and Patterns during Pregnancy

Women either maintained or changed their eating behaviors when pregnant (Table 1). Briefly, most women (77%) reported an increased intake of a daily vitamin during pregnancy. Most also reported (i) snacking more frequently (72%), especially closer to bed time (40.5%); (ii) eating more (60.9%), but with similar night-time eating (63.9%); and (iii) not counting calories as often as pre-pregnancy (50.8%). However, they reported maintaining the amount of fat (64%), salty foods (56.5%), and fried or fast foods (47.1%) consumed during pregnancy. 

Despite these pregnancy-related dietary changes, the majority of women denied trying a specific dietary pattern during pregnancy (84.9 to 95.1% across diets), and most reported being unwilling to try a vegan (81%), vegetarian (70.7%), or Paleo, Ketogenic, Atkins, or similar low-carb diet (66.6%) (Table 2). There were no relationships between women who had previously tried a specific dietary pattern outside of pregnancy and willingness to try a dietary pattern during pregnancy (*p* > 0.05). Among the few who had tried or were willing to try a dietary pattern, a woman described, “I’ve been a vegetarian for 10 years, and I maintained the vegetarian diet through both of my 2 pregnancies”. Another stated, “I am really open to trying these! And planning to get pregnant again soon!”

In examining whether attempts or willingness to try dietary patterns were related to perceptions of safety, notably we found that women were willing to try any dietary pattern if they deemed it safe during pregnancy (*p* < 0.05). Specifically, almost all women deemed low- or no sugar foods (94.9%), a Mediterranean diet (84.5%), and the Whole-30 diet (81.6%) to be safe during pregnancy, while almost half rated (46.1%) calorie-counting as unsafe (Table 2). A woman reported, “In my opinion, I honestly think no pregnant woman should be dieting or losing weight while pregnant. It can be unsafe for the unborn child and mother”. Another stated, “In my opinion, I do not think pregnant women should be dieting at all unless you are already obese when you become pregnant”.

Concerns for following a vegetarian/plant-based dietary pattern or a Paleo, Ketogenic, Atkins, or a similar low-carb diet included the requirement to eat a balanced diet (65.9% and 60.1% respectively), difficultly to follow without family (64.8% and 63.2% respectively), and expense (58.7% and 60.1% respectively) (Table 3). On the topic of balanced diets, a woman expressed, “Pregnancy is a time to eat a well-balanced diet, not a time for restrictive fad diets”.

Periodic consultation with a dietitian or nutritionist (85% and 86.7% respectively) and being provided all meals and snacks (88.1% and 90.6% respectively) were perceived as helpful strategies by most women to follow both a vegetarian/plant-based dietary pattern or a Paleo, Ketogenic, Atkins, or similar low-carb diet (Table 4). A woman shared, “Diets that restrict any macronutrients is taking away nutrients that is needed to build a healthy baby and replenish nutrients that is leaving the mother. To be able to follow diets that don’t eat animal products would require constant monitoring of food, vitamins, and bloodwork to ensure proper nutritional needs are being met”.

### 3.3. Eating Behaviors and Patterns by Parity, Weight Status, and Trimester

#### 3.3.1. Parity

Eating behaviors. When examining responses by parity, we found nulliparous women, compared to multiparous women, to be 57% and 50% less likely, respectively, to increase (vs. decrease) (0.43 [CI 0.22–0.85], *p* = 0.016) or maintain (vs. decrease) (0.50 [CI 0.28–0.89], *p* = 0.018) the amount of fat. The latter was partly determined by Caucasian race (2.54 [CI 1.34–4.80], *p* = 0.004).

Dietary patterns. Nulliparous women were 56% more likely to have tried a vegetarian diet during pregnancy compared to multiparous women (0.44 [CI 0.22–0.88], *p* = 0.019). This was partly determined by Caucasian race (3.61 [CI 1.77–7.35], *p* < 0.0001) and non-married status (0.16 [CI 0.036–0.72], *p* = 0.017).

#### 3.3.2. Weight Status

Eating behaviors. Compared to normal-weight women, women with obesity were more likely to adopt healthy dietary behaviors (vs. maintain the same pre-pregnancy behaviors), including improving the healthfulness of food consumed (2.07 [CI 1.31–3.29], *p* = 0.002) as well as decreasing the size of meal (0.51 [CI 0.30–0.86], *p* = 0.012) and amount of food (0.29 [CI 0.12–0.63], *p* = 0.002), including fried food (0.55 [CI 0.35–0.88], *p* = 0.012), fat (0.45 [CI 0.24–0.85], *p* = 0.014), and snacking frequency (0.34 [CI 0.14–0.84], *p* = 0.019). However, they were more likely to decrease fruit consumption (0.34 [CI 0.12–0.93], *p* = 0.036). Higher education and Caucasian race partly determined some of these relationships. Additionally, compared to normal-weight women, women with overweight were more likely to increase (vs. maintain) the amount of vegetables consumed (1.72 [CI 1.11–2.67], *p* = 0.015). Please refer to Supplementary Table S1 for more details. 

Dietary patterns. Compared to normal-weight women, women with obesity were less likely to have tried a Mediterranean diet (4.23 [CI 1.20–14.9], *p* = 0.025). Yet, they were more likely to have tried a low-carb diet (0.31 [CI 0.15–0.64], *p* = 0.002). Women with obesity were also more willing to try a low-carb diet (0.60 [CI 0.37–0.98], *p* = 0.043) compared with normal-weight women. Compared to normal-weight women, women with overweight were more likely to have tried a low-carb diet (0.29 [CI 0.14–0.60], *p* = 0.001) as well as a Whole-30 diet (0.28 [CI 0.12–0.69], *p* = 0.006). Higher education was a partial determinant of the likelihood of having tried a low-carb diet among those in the overweight and obesity groups (0.46 [CI 0.24–0.89], *p* = 0.021).

With regard to safety, women with overweight were more likely to consider a Whole-30 diet safe (0.44 [CI 0.20–0.96], *p* = 0.038) compared to normal-weight women. This was partly determined by younger age (2.36 [CI 1.24–4.47], *p* = 0.009) and non-married status (0.32 [CI 0.13–0.77], *p* = 0.11).

Women with obesity had several concerns in relation to following a vegetarian diet. Compared to normal-weight women, women with obesity were more likely to be concerned by following a vegetarian diet due to price (0.47 [CI 0.27–0.83], *p* = 0.009), having a busy schedule (0.43 [CI 0.25–0.74], *p* = 0.002), hunger (0.49 [CI 0.29–0.84], *p* = 0.010), compromised enjoyment (0.38 [CI 0.22–0.66], *p* = 0.001), and not eating enough (0.38 [CI 0.22–0.66], *p* = 0.001). Price concerns among women with obesity were partly determined by higher education (0.53 [CI 0.33–0.86], *p* = 0.010).

Some strategies may help women with overweight and obesity to follow a low-carb or vegetarian diet. Compared to normal-weight women, women with obesity were more likely to consider dietetic consultations helpful to adopt a low-carb diet (0.39 [CI 0.17–0.94], *p* = 0.035). As for women with overweight, they were more likely to consider adhering to a diet on five days helpful to adopt a low-carb diet (0.49 [CI 0.25–0.96], *p* = 0.038) and a vegetarian diet (0.48 [CI 0.25–0.92], *p* = 0.027) compared to normal-weight women. These relationships were partly determined by non-married status (0.40 [CI 0.20–0.82], *p* = 0.012 and 0.47 [CI 0.24–0.95], *p* = 0.034, respectively). 

#### 3.3.3. Trimester

Eating behaviors. Compared to women in the third trimester, women in the first trimester were less likely to increase (vs. maintain) the amount of food consumed (0.38 [CI 0.18–0.81], *p* = 0.011) and less likely to increase (vs. decrease) (0.33 [CI 0.14–0.81], *p* = 0.015) the amount of food consumed. The latter was partly determined by higher education (0.29 [CI 0.13–0.68], *p* = 0.004). Compared to women in the third trimester, women in the first trimester were also less likely to maintain (vs. decrease) the amount of sugar consumed (0.43 [CI 0.20–0.91], *p* = 0.028) and were less likely to increase (vs. decrease) the amount of sugar consumed (0.35 [CI 0.15–0.84], *p* = 0.019).

Compared to women in the third trimester, women in the second trimester were more likely to increase (vs. decrease) the amount of fruit consumed (4.39 [CI 1.31–14.7], *p* = 0.017), more likely to increase (vs. maintain) the amount of salty foods consumed (2.69 [CI 1.51–4.77], *p* = 0.001), and more likely to increase (vs. decrease) the amount of salty foods consumed (2.77 [CI 1.31–5.83], *p* = 0.007). The latter was partly determined by Caucasian race (3.31 [CI 1.45–7.56], *p* = 0.005).

Dietary patterns. Compared to women in the third trimester, women in the second trimester were less likely to consider a vegan diet to be safe (2.0 [CI 1.08–3.68], *p* = 0.027). 

In addition, they were more likely to be concerned in following a vegetarian diet due to hunger (0.56 [0.32–0.98], *p* = 0.044), own health (0.55 [CI 0.31–0.97], *p* = 0.040), and baby’s health (0.47 [0.27–0.83], *p* = 0.009), which was partly determined by non-Caucasian race (0.43 [CI 0.21–0.89], *p* = 0.023). Compared to women in the third trimester, women in the first trimester were more likely to be concerned in following a low-carb diet due to price (2.63 [CI 1.12–6.18], *p* = 0.027), which was determined by higher education (0.41 [CI 0.23–0.74], *p* = 0.003).

### 3.4. General Perceptions of Eating Behaviors and Patterns during Pregnancy

Seventy women who completed the long-form of the survey provided additional information in free-text comment boxes. Seven categories emerged: perceptions of dieting during and outside of pregnancy, influence of pre-existing dietary habits, influence of dietary preferences during pregnancy, intersection between dietary patterns and adequate nutrition, intersection between dietary patterns and underlying medical conditions, adherence to evidence-based guidelines and recommendations, and guidance from health care professionals (exemplar quotes in Table 5 and embedded in text above). Briefly, many women expressed a reluctance to follow any restrictive diets during pregnancy due to perceived inadequate nutrient and energy intake, and subsequent harms for the mother and baby. Women’s pre-existing dietary habits, as well as food preferences, shaped their perceptions towards trying the proposed dietary patterns during pregnancy. Perceptions were particularly altered in the case of existing medical conditions or diseases during pregnancy. Most conditions deterred women from adopting certain dietary patterns except in the case of obesity, where few women reported benefiting from monitoring dietary behaviors and weight loss. As was similarly observed in our quantitative findings, some women were receptive to certain dietary patterns with proper, professional support such as from dietitians, and in the case of strong evidence-based research.

## 4. Discussion

This was the first study to explore women’s perceptions of eating behaviors and modern dietary patterns during pregnancy. Our quantitative and quasi-qualitative findings revealed that women may be apprehensive towards following a specific dietary pattern during pregnancy, especially since most dietary patterns were perceived to be restrictive and not well-balanced. Notable exceptions appeared in the case of pre-existing medical conditions (e.g., obesity), in which case certain diets were recommended, perceived to be beneficial, and implemented more often in women with obesity compared to normal-weight women. Specifically, women with obesity in our study reported making healthier dietary changes, with the exception of fruit intake, compared to normal-weight women, even though they had not previously attempted to follow a specific dietary pattern and had heightened hesitations towards trying new diets. These changes may be influenced by time point of pregnancy, as we also show that women in the first trimester appeared to be more likely to follow healthier eating behaviors than women in late pregnancy. Longitudinal data comparing the same women at each time point can further elucidate this relationship. Interestingly, the number of pregnancies did not fully dictate eating behaviors and patterns of women, suggesting that prior experiences of being pregnant did not alter women’s perceptions or behaviors surrounding diet. Our findings challenge the notion of pregnancy as a standardized, or one-size-fits-all teachable moment. Rather, our study underscores the importance of tailoring care to individual needs, characteristics, and circumstances, which highlights the importance of early, personalized interventions to optimize healthy dietary behavior for every pregnancy. 

The dietary patterns explored within this study are prevalent in popular culture. While ‘dieting’ is often associated with restrictive behavior, certain dietary patterns can offer many health benefits with proper guidance. Some, like the Mediterranean diet, have been studied extensively in non-pregnant populations. For example, in a systematic review and meta-analysis, the Mediterranean diet has shown favorable effects on body composition (body weight, BMI, waist circumference), systolic and diastolic blood pressure, glycemic health (glucose, insulin, homeostatic model assessment of insulin resistance index), lipids (total cholesterol, low-density lipoprotein cholesterol, high-density lipoprotein cholesterol, triglycerides), and inflammatory markers (C-reactive protein, interleukin-6, tumour necrosis factor-a) compared to no treatment, usual care, or different diets in adults [25]. Physiological changes during pregnancy are marked by insulin resistance and increased lipid availability, traits which are further exacerbated by pre-existing metabolic disorders, such as maternal obesity. However, according to the 2020–2025 Dietary Guidelines for Americans, there is no research to support any of the proposed diets for added benefits in pregnancy [3]. Conducting research in this population may be challenging with preconceived notions that any diet in pregnancy is likely restrictive. Therefore, identifying non-restrictive dietary patterns that may similarly offer benefits to pregnant women, while being personalised to their individual characteristics, is crucial. Communicating the potential benefits of such diets to women will be an important step for increased implementation and adherence.

Understanding women’s eating behaviors and dietary patterns is the first step to inform potential dietary interventions in pregnancy. In addition to increased nutrient and energy needs, differences in food choice between pregnant and non-pregnant women pertain to the avoidance of potentially harmful food groups [26]. However, many women are unaware of food safety guidelines [27], and few achieve optimum knowledge about healthy eating guidelines for pregnancy [28]. Previous reports have shown that many women did not change the amount and types of foods consumed during pregnancy and that the consumption of fish, vegetables, and other foods remained as low as the pre-pregnancy state [21,22]. However, others have shown that women adopt altered dietary changes after becoming pregnant, including consuming more fruit, vegetables, dairy, high-fiber foods, and fish [28,29]. These studies did not examine changes by subgroups, so the overall effects from individual characteristics cannot be disentangled. While the present study did not examine specific food groups, we found that dietary patterns varied with individual characteristics (e.g., sociodemographics, trimester, weight status). 

Similar to our findings, pregnant women who had overweight and obesity in previous research were found to be less likely than normal-weight women to achieve recommended fruit intakes [30]. However, this study found that these women were more likely to adopt unhealthy eating behaviors than normal-weight women, including consumption of sugar-sweetened beverages and take away foods [30]. These findings are contrary to reports in our study where women with obesity reported adopting overall healthier dietary changes than normal-weight women. Of note, dietary quality in pregnant women with and without overweight and obesity has been shown to decrease across trimesters in previous research and in our study, respectively [31]. However, examinations of maternal dietary patterns during each trimester of pregnancy revealed that the majority of women with an ‘unhealthy’ or ‘health conscious’ dietary pattern in early pregnancy remained in their respective groups across all trimesters [32]. These observations indicate that dietary behaviors of pregnant women, including women with overweight and obesity, may fluctuate at different time points of pregnancy despite the overall dietary pattern followed. We were not able to examine weight status by trimester interactions due to limitations in sample size. However, given previous observations, early interventions focusing on individual needs and circumstances is warranted.

In our cohort of pregnant women, it is plausible that women with obesity were less likely to try or be willing to try new diets given the overwhelming amount of circulating dietary information and uncertainties surrounding the most effective approach for weight management and pregnancy health. Professional support in these cases may be instrumental to clarify any misconceptions and to promote healthy dietary patterns, especially considering that (i) recent changes to dietary intake recommendations throughout pregnancy are not tailored to pregnant women with obesity [3] and (ii) the IOM guidelines for women with obesity are challenged by the research community and may overestimate energy needs [33]. As evidenced in our study, women overall and across all groups, especially women with obesity, desired professional support from a trusted source such as a dietitian when attempting new diets. While women are in frequent contact with their prenatal care provider, conversations around weight-related behavioral counseling are self-initiated nearly half the time [34] and are insufficient to address their challenges or misconceptions in relation to adequate nutrition [15,28,35,36]. Furthermore, a limited number of pregnant women follow advice from a dietitian [37]. Health care professionals have particularly expressed communication difficulties around weight due to perceptions of obesity being a sensitive and stigmatizing issue [38]. In agreement with a recent statement by the US Preventative Task Force, this indicates that interventions should focus on educating and supporting health care professionals in working with this population, alongside a focus on personalized care and providing unambiguous and appropriate pregnancy-related dietary information and behavioral counseling [38,39]. Importantly, pregnant women desire positive health messages around healthy eating and weight management during pregnancy, with a focus on what they can/should do, as opposed to what they should not do [40]. Advice can be incorporated into a locus of control model, whereby shifting health beliefs and health locus of control from external causes to internal dimensions may support healthy behavioral tendencies [41]. 

Alongside our study’s strengths, including a large sample size and personalized approach, there are several limitations to be acknowledged. Most of our participants were married, Caucasian women. While we adjusted for marital status and race in our analyses, the generalizability of our findings is inherently limited to this sample. In addition, while there are many indicators of socioeconomic status, such as education, we did not collect data on income. We also did not collect data on pre-pregnancy dietary habits and pre-existing comorbidities other than obesity. As such, some of our findings may have been driven by income, dietary regimens followed outside of pregnancy, or medical conditions, which are important points of consideration in devising dietary interventions. To minimize participant burden, we also only collected data on concerns and helpful strategies in following a vegetarian/plant-based diet as well as a low-carb diet. This calls for examining subgroup differences across several other diets that women may be interested in following if proven beneficial. In addition, survey studies are prone to the inherent limitations of self-reported data, meaning that reported changes to eating behaviors and patterns may not fully correlate with observational data. Interpretation of the survey questions and terminology is also prone to subjectivity. We observed this in how women interpreted ‘diet’ to mean ‘restriction’ in most cases, despite some of our questions deliberately using terminology such as “eating patterns”. As definitions of the diets were not provided, women may have also interpreted these differently. Finally, responses from participants using a free-text may have been indirectly shaped by closed-ended questions, such as our agenda as researchers [42]. These data can only be seen as quasi-qualitative as they do not possess the strength of a qualitative study; that is, conceptual richness, relevance to context, and depth of responses. Despite these limitations, women provided detailed comments, most of which were attached with an emotional response not captured by the quantitative date, thereby prompting a formal analysis [42].

## 5. Conclusions

Together, our study challenges the notion that there is a universal equation to instilling teachable moments during pregnancy. Women may not be averse to change; however they would benefit from understanding how certain dietary changes may be beneficial to their health and their infants’ health in the absence of a caloric deficit. In providing services and devising interventions to pregnant women, care must be sought to personalize teachable moments based on women’s individual characteristics and circumstances. Importantly, early interventions are warranted to connect with women most adaptable to change. Sociodemographic factors (age, race, education, marital status) and others, such as parity, weight status, and trimester, may further alter perceptions and behaviors and should be considered. In addition, many women diet before and during pregnancy, which may further exacerbate suboptimal dietary practices in the first trimester and beyond. Health care professionals, including dietitians, are encouraged to consider these factors on an individual level and may benefit from capitalizing on their communication skills to ensure that information that is shared with women is coherent, unequivocal, and fosters a motivation for change.

## Figures and Tables

**Table 1:** Eating behavior changes during pregnancy.

Eating Behaviors ^a^	Less (%)	Same (%)	More (%)
Taking a daily vitamin	3.3	19.7	77
Healthfulness of food	11.2	38	50.8
Amount of food	12.1	27.1	60.9
Size of meals	26	40.7	33.3
Amount of sugar	25.3	45.6	29.1
Amount of fat	12.7	64	23.2
Amount of salty foods	17.3	56.5	26.1
Amount of fried or fast foods	36.1	47.1	16.8
Amount of fruit	5.1	36.4	58.5
Amount of vegetables	8.2	45.8	46
Amount of fiber	5.2	56.3	38.4
Time between last meal/snack and bed time	40.5	41.6	17.9
Night-time eating/drinking	11.6	63.9	24.5
Snacking frequency	7.3	20.7	72
Breakfast frequency	6.6	50.2	43.2
Sugar-sweetened beverage frequency	33.7	48.4	17.9
Calorie-counting frequency	50.8	45.2	4

^a^ *n* = 522–547 across items. Responses were not required for each of the items. Women were asked: Compared to before you were pregnant, how would you rate your changes to the following eating behaviors when pregnant? (If you are currently not pregnant, please answer for when you were pregnant). Available responses were: A lot less; A little less; About the same; A little more; A lot more; and I don’t know. A lot less and A little less were categorized into Less whereas A little more and A lot more were categorized into More.

**Table 2:** Perceptions toward modern dietary patterns during pregnancy.

Dietary Patterns ^a^	Tried (%)	Not Tried (%)	Willing (%)	Not Willing (%)	Safe (%)	Not Safe (%)
Paleo, Ketogenic, Atkins, or similar low-carb diet	12.8	87.2	33.4	66.6	64.2	35.8
Mediterranean or DASH diet	6.5	93.5	45.4	54.6	84.5	15.5
Whole-30 diet	5.9	94.1	48.4	51.6	81.6	18.4
Vegetarian diet	8.7	91.3	29.3	70.7	74.5	25.5
Vegan diet	4.9	95.1	19	81	60.7	39.3
Calorie-counting	15.1	84.9	42	58	53.9	46.1
Low-fat foods	-	-		-	78.3	21.7
Low-carb foods	-	-		-	79.9	20.1
Low- or no sugar foods	-	-		-	94.9	5.1

^a^ *n* = 291–510 across domains and items. Responses were not required for each of the items. Women were asked: When pregnant, have you followed any of these diets? If not, would you be willing to work with a dietician to follow one of these dietary patterns during the second or third trimester of pregnancy? Available responses were: I have tried this diet during pregnancy; I have tried this diet during pregnancy and did not like it; I have not tried this diet and would not be willing to try it during my pregnancy; and I have not tried this diet but would be willing to try it during my pregnancy. Women who tried or were willing to try any of the diets were categorized as Tried and Willing, respectively, whereas those who have not tried or were not willing to try any of the diets were categorized as Not tried and Not willing, respectively. Women were also asked: How safe would you rate following these diets during your second and third trimester of pregnancy? Available responses were: Not at all safe, Not very safe; Somewhat safe; Very Safe; and I don’t know. Not at all safe and Not very safe were categorized as Not safe, whereas Somewhat safe and Very Safe were categorized as Safe.

**Table 3:** Concerns about following dietary patterns during pregnancy.

	Vegetarian/Plant-Based		Paleo, Ketogenic, Atkins, or Similar Low-Carb	
Reason ^a^	Concern (%)	Not a Concern (%)	Concern (%)	Not a Concern (%)
I would not enjoy the food	56.2	43.8	44.9	55.1
I would be too hungry	57.2	42.8	49.7	50.3
I would get nauseous	46.9	53.1	49.4	50.6
I would not eat enough food	54	46	44.3	55.7
It would be hard to eat a balanced diet	65.9	34.1	60.1	39.9
It would not be safe for my health	43.8	56.2	46.5	53.5
It would not be safe for my baby’s health	55.1	44.9	55.4	44.6
It would not work with my busy schedule	39.6	60.4	38.8	61.2
It would be expensive	58.7	41.3	60.1	39.9
It would be hard to follow if my family doesn’t join me	64.8	35.2	63.2	36.8

^a^ *n* = 360–361 across domains and items. Responses were not required for each of the items. Women were asked: What concerns would you have about following these eating plans during your second or third trimester of pregnancy? Available responses were: Concern for Vegetarian/Plant-based diet; Concern for Paleo, Ketogenic, Atkins, or similar low-carb diet; Concern for both of these diets; and Not a concern for either of these diets.

**Table 4:** Helpful strategies to follow dietary patterns during pregnancy.

	Vegetarian/Plant-Based		Paleo, Ketogenic, Atkins, or Similar Low-Carb	
Strategy ^a^	Helpful (%)	Not Helpful (%)	Helpful (%)	Not Helpful (%)
Being provided protein shakes or bars to supplement my own diet	77.6	22.4	79.2	20.8
Being provided all of my meals and snacks	88.1	11.9	90.6	9.4
Following this diet on only five days of the week	77.6	22.4	80.3	19.7
Meeting with a dietitian or nutritionist periodically	85	15	86.7	13.3
Checking in with my doctor more often to make sure my baby is healthy	82.5	17.5	85.3	14.7

^a^ *n* = 361. Responses were not required for each of the items. Women were asked: Select if these things would make following each diet more helpful. Available responses were: Helpful for Vegetarian/Plant-based diet; Helpful for Paleo, Ketogenic, Atkins, or similar low-carb diet; Helpful for both of these diets; and Not helpful for either of these diets.

**Table 5:** Supporting quotes of women’s perceptions in relation to eating behaviors and patterns during pregnancy.

Category ^a^	Quotes
**Perceptions of dieting** **during and outside of** **pregnancy**	“I don’t really believe sticking to a specific diet plan is realistic during or outside of pregnancy regardless of health benefits or risks.”
	“Pregnancy is a time to eat a well-balanced diet, not a time for restrictive fad diets.”
	“In my opinion, I honestly think no pregnant woman should be dieting or losing weight while pregnant. It can be unsafe for the unborn child and mother.”
	“I generally feel like elimination or restriction diets are not a good idea, particularly during pregnancy. I think moderation and variety are the most important factors in a diet, although I recognize that my diet is not necessarily ideal currently.”
	“The very idea of dieting while pregnant sounds crazy and dangerous to me. It’s important to maintain physical fitness and to gain weight slowly, but it is not a time to try and lose weight.”
**Influence of pre-existing** **dietary habits**	“I work overnights and don’t have a consistent schedule for sleeping and eating. Also, my husband is already vegan and makes most of our meals, I eat dairy snacks and very occasionally eat meat. A low carb diet instead would be really hard to follow.”
	“I’ve been a vegetarian for 10 years, and I maintained the vegetarian diet through both of my 2 pregnancies.”
	“I was doing the Keto diet from July 2020- Sept 2020 and stopped when I learned I was pregnant due to so many cravings outside the diet. It was challenging but it is a diet I would consider starting again postpartum. This diet I think, although doable, would be challenging to overcome cravings outside of the diet. My self-control is out the window when I am pregnant!”
	“I eat the same way pregnant as not, but I am much hungrier pregnant and eat to satiety and to support an extremely active lifestyle. I put on the weight gradually and lose it gradually, and I think it’s what my body needs to do to build and breastfeed babies!”
**Influence of dietary** **preferences during pregnancy**	“My concern for plant-based/vegetarian is that I do not usually like the types of fats and proteins available in those diets—so it is a food preference issue.”
	“I prefer lean meat which includes fish.”
	“I am really open to trying these! And planning to get pregnant again soon!”
**Intersection between dietary patterns and adequate** **nutrition**	“Diets that restrict any macronutrients is taking away nutrients that is needed to build a healthy baby and replenish nutrients that is leaving the mother. To be able to follow diets that don’t eat animal products would require constant monitoring of food, vitamins, and bloodwork to ensure proper nutritional needs are being met.”
	“I would be concerned with a vegetarian or vegan diet because I think I would have low iron and iron supplements make me very sick while pregnant, vomit for hours.”
	“I don’t think vegan is a good diet for pregnancy because you need DHA and omega 3 from fish.”
	“Many foods on the Keto diet I am allergic to (dairy) and I do not believe that eating bacon is better for you than eating a sweet potato.”
**Intersection between dietary patterns and underlying medical conditions**	“I have celiac disease so the vegetarian diet would be more concerning as far as getting enough nutrients”
	“I’ve had issues with gallbladder during pregnancy and started being able to tolerate eating when I was mindful of reducing fat sources and increasing simple carb snacks.”
	“I did a low carb diet after 28 weeks with my first pregnancy, due to Gestational Diabetes.”
	“In my opinion, I do not think pregnant women should be dieting at all unless you are already obese when you become pregnant.”
	“I did not follow anything specific but being in the obese category I just watched my intake and drank more water than usual—and no other beverages really. I only gained 17 lbs by the end of my pregnancy and I was aiming for 15–20.”
	“I personally have very bad pregnancy sickness and would be unable to have any sort of dietary restrictions. The thought of eating gives me major anxiety. To be honest, I often choose my meals based on what will taste the best and cause the least burning in my esophagus when regurgitated.”
	“I haven’t had issues with weight gain or blood pressure during either of my pregnancies so maybe I’m coming from a different place since I mostly eat when and what I want.”
**Adherence to evidence-based guidelines and recommendations**	“I found it helpful to follow my regular, generally healthy eating style, and not obsessively worry too much about ‘foods you shouldn’t eat during pregnancy.’ I just focused on getting enough Whole Foods from different food groups. I didn’t eat fish high in mercury, and avoided undercooked eggs/meat and unpasteurized dairy, but also not obsessively.”
	“If I’m at a normal weight and exercise regularly, there isn’t much incentive for me to introduce a new diet program unless there was strong evidence it was better for me and the baby.”
	“One needs to be careful about the mercury content in fish and avoid the ones that have high mercury content. Intake of fibers are very important because it’s not just healthy when you are pregnant, it also helps with alleviating constipation during pregnancy. Dates, figs if taken in moderation serve as very good snacks. A glass of milk (lactose free if you’re lactose intolerant) before bed worked wonders for me to keep my night time hunger at bay and for calcium.”
**Guidance from health care professionals**	“I do not know enough about these diet programs to know if they are safe during pregnancy. I assume that they are but I would personally want to consult with my Dr and a nutritionist if I were going to go on a special diet during pregnancy.”
	“My biggest concern with some of the diets listed (Paleo, Keto, Atkins) is the restriction of carbohydrates which is the primary energy source for both mom and baby. I believe these diets can be followed while pregnant BUT with clear guidance and monitoring by a dietitian to ensure energy needs are met.”
	“I worked with a metabolic specialist for years leading up to my pregnancy and through my pregnancy, and they teach that any type of restriction-based dieting has severe long-term effects on the metabolism and absolutely should not happen during pregnancy. I wouldn’t trust any doctor who recommended a diet during pregnancy.”

^a^ *n* = 70. Women were provided with an open-ended text box to provide any additional details regarding their thoughts on dietary and physical activity habits during pregnancy.

## Data Availability

The data presented in this study are available on request from the corresponding author.

## Supplementary Materials

The following are available online at https://www.mdpi.com/article/10.3390/nu13093298/s1, Table S1: Significant relationships for women with obesity compared to normal-weight women; Survey.
