# Supplementary material for: Eating Behaviors and Dietary Patterns of Women during Pregnancy: Optimizing the Universal ‘Teachable Moment’
Source: Nutrients. 2021 Sep 21;13(9):3298. doi: 10.3390/nu13093298 (PMC8471126; doi:10.3390/nu13093298)
Supplement: Supplementary file 1 [file nutrients-13-03298-s001.zip › Modern Mommy Bites_Survey Questions_IRB Approved 11122020.pdf]

Modern Mommy Bites: Eating and exercise behaviors of moms during pregnancy

The purpose of this survey is to see how mothers feel about certain diet and exercise behaviors during pregnancy. Please answer the following questions based on your opinion.

Your participation in this survey is voluntary, and you do not have to participate if you do not want to. If at any time you are uncomfortable with answering any question, you do not have to submit the survey. By submitting this survey, you provide your consent for our research team to review and use the information you share with us. Your responses will be kept confidential.

If you would like to enter the draw for one of ten $50 for your participation, you can choose to provide your email address at the end of the survey. This is optional and not required for participation. Thank you.

- o I agree to continue the survey.
- o I do not agree to continue the survey.

If "I do not agree to continue the survey" is selected, the survey displays the following verbiage:
End the survey?

You have selected an option that triggers this survey to end right now. To save your responses and end the survey, click the button below to do so. If you have selected the wrong option by accident and do not wish to leave the survey, you may click the other button below to continue, which will also remove the value of the option you just selected to allow you to enter it again and continue the survey.

Continue survey and undo last response **End the survey now**

1. What is your sex?
   - o Female
   - o Male

If "Male" is selected to question 1, the survey displays the following verbiage:
End the survey?

You have selected an option that triggers this survey to end right now. To save your responses and end the survey, click the button below to do so. If you have selected the wrong option by accident and do not wish to leave the survey, you may click the other button below to continue, which will also remove the value of the option you just selected to allow you to enter it again and continue the survey.

Continue survey and undo last response **End the survey now**

2. What is your age?
   - o Under 18 years
   - o 18-24 years
   - o 25-30 years

- 31-35 years
- 36-40 years
- 41 years and over

If "Under 18 years" is selected to question 2, the survey displays the following verbiage:
End the survey?

You have selected an option that triggers this survey to end right now. To save your responses and end the survey, click the button below to do so. If you have selected the wrong option by accident and do not wish to leave the survey, you may click the other button below to continue, which will also remove the value of the option you just selected to allow you to enter it again and continue the survey.

Continue survey and undo last response **End the survey now**

3. Where do you live?
   - Africa
   - Asia
   - Australia
   - Canada
   - Europe
   - South America
   - Mexico
   - United Kingdom
   - United States of America

If any option other than "United States of America" is selected to question 3, the survey displays the following verbiage:
End the survey?

You have selected an option that triggers this survey to end right now. To save your responses and end the survey, click the button below to do so. If you have selected the wrong option by accident and do not wish to leave the survey, you may click the other button below to continue, which will also remove the value of the option you just selected to allow you to enter it again and continue the survey.

Continue survey and undo last response **End the survey now**

4. Choose what best describes your pregnancy status.
   - I am pregnant now AND have given birth in the past two years
   - I am pregnant now but have NOT given birth in the past two years
   - I am NOT pregnant now but HAVE given birth in the past two years
   - I am NOT pregnant now and have NOT given birth in the past two years

If "I am NOT pregnant now and have NOT given birth in the past two years" is selected to question 4, the survey displays the following verbiage:
End the survey?

You have selected an option that triggers this survey to end right now. To save your responses and end the survey, click the button below to do so. If you have selected the wrong option by accident and do not wish to leave the survey, you may click the other button below to

continue, which will also remove the value of the option you just selected to allow you to enter it again and continue the survey.

Continue survey and undo last response **End the survey now**

If "I am pregnant now AND have given birth in the past two years" is selected to question 4, the survey doesn't display question 6.

If "I am NOT pregnant now but HAVE given birth in the past two years" is selected to question 4, the survey doesn't display questions 5, 6, and 9.

NEXT PAGE

15% Completed [progress bar]

In this section, we will ask you to tell us about yourself.

5. How many weeks pregnant are you?
   - ○ Less than 6 weeks
   - ○ 6
   - ○ 7
   - ○ 8
   - ○ 9
   - ○ 10
   - ○ 11
   - ○ 12
   - ○ 13
   - ○ 14
   - ○ 15
   - ○ 16
   - ○ 17
   - ○ 18
   - ○ 19
   - ○ 20
   - ○ 21
   - ○ 22
   - ○ 23
   - ○ 24
   - ○ 25
   - ○ 26
   - ○ 27
   - ○ 28
   - ○ 29
   - ○ 30
   - ○ 31
   - ○ 32
   - ○ 33
   - ○ 34
   - ○ 35
   - ○ 36
   - ○ 37

- 38
- 39
- 40
- 41

6. Is this your first pregnancy, or your first pregnancy that lasted past 12 weeks?
   - Yes
   - No

7. How many children do you have excluding your current pregnancy (if applicable)?
   - 0
   - 1
   - 2
   - 3
   - 4
   - 5 or more

If “0” is selected to question 7, the survey doesn’t display second last item in question 33 and last item in question 34.

8. How much do you weigh (in pounds)?
   - Less than 100 lbs
   - 101 lbs
   - 102 lbs
   - 103 lbs
   - 104 lbs
   - 105 lbs
   - 106 lbs
   - 107 lbs
   - 108 lbs
   - 109 lbs
   - 200 lbs
   - 201 lbs
   - 202 lbs
   - 203 lbs
   - 204 lbs
   - 205 lbs
   - 206 lbs
   - 207 lbs
   - 208 lbs
   - 209 lbs
   - 210 lbs
   - 211 lbs
   - 212 lbs
   - 213 lbs
   - 214 lbs
   - 215 lbs
   - 216 lbs
   - 217 lbs
   - 218 lbs
   - 219 lbs

- 220 lbs
- 221 lbs
- 222 lbs
- 223 lbs
- 224 lbs
- 225 lbs
- 226 lbs
- 227 lbs
- 228 lbs
- 229 lbs
- 230 lbs
- 231 lbs
- 232 lbs
- 233 lbs
- 234 lbs
- 235 lbs
- 236 lbs
- 237 lbs
- 238 lbs
- 239 lbs
- 240 lbs
- 241 lbs
- 242 lbs
- 243 lbs
- 244 lbs
- 245 lbs
- 246 lbs
- 247 lbs
- 248 lbs
- 249 lbs
- 250 lbs
- 251 lbs
- 252 lbs
- 253 lbs
- 254 lbs
- 255 lbs
- 256 lbs
- 257 lbs
- 258 lbs
- 259 lbs
- 260 lbs
- 261 lbs
- 262 lbs
- 263 lbs
- 264 lbs
- 265 lbs
- 266 lbs
- 267 lbs
- 268 lbs
- 269 lbs
- 270 lbs

- 271 lbs
- 272 lbs
- 273 lbs
- 274 lbs
- 275 lbs
- 276 lbs
- 277 lbs
- 278 lbs
- 279 lbs
- 280 lbs
- 281 lbs
- 282 lbs
- 283 lbs
- 284 lbs
- 285 lbs
- 286 lbs
- 287 lbs
- 288 lbs
- 289 lbs
- 290 lbs
- 291 lbs
- 292 lbs
- 293 lbs
- 294 lbs
- 295 lbs
- 296 lbs
- 297 lbs
- 298 lbs
- 299 lbs
- 300 lbs
- More than 300 lbs

9. How much did you weigh before you were pregnant (in pounds)?
   - Less than 100 lbs
   - 101 lbs
   - 102 lbs
   - 103 lbs
   - 104 lbs
   - 105 lbs
   - 106 lbs
   - 107 lbs
   - 108 lbs
   - 109 lbs
   - 200 lbs
   - 201 lbs
   - 202 lbs
   - 203 lbs
   - 204 lbs
   - 205 lbs
   - 206 lbs
   - 207 lbs

- 208 lbs
- 209 lbs
- 210 lbs
- 211 lbs
- 212 lbs
- 213 lbs
- 214 lbs
- 215 lbs
- 216 lbs
- 217 lbs
- 218 lbs
- 219 lbs
- 220 lbs
- 221 lbs
- 222 lbs
- 223 lbs
- 224 lbs
- 225 lbs
- 226 lbs
- 227 lbs
- 228 lbs
- 229 lbs
- 230 lbs
- 231 lbs
- 232 lbs
- 233 lbs
- 234 lbs
- 235 lbs
- 236 lbs
- 237 lbs
- 238 lbs
- 239 lbs
- 240 lbs
- 241 lbs
- 242 lbs
- 243 lbs
- 244 lbs
- 245 lbs
- 246 lbs
- 247 lbs
- 248 lbs
- 249 lbs
- 250 lbs
- 251 lbs
- 252 lbs
- 253 lbs
- 254 lbs
- 255 lbs
- 256 lbs
- 257 lbs
- 258 lbs

- 259 lbs
- 260 lbs
- 261 lbs
- 262 lbs
- 263 lbs
- 264 lbs
- 265 lbs
- 266 lbs
- 267 lbs
- 268 lbs
- 269 lbs
- 270 lbs
- 271 lbs
- 272 lbs
- 273 lbs
- 274 lbs
- 275 lbs
- 276 lbs
- 277 lbs
- 278 lbs
- 279 lbs
- 280 lbs
- 281 lbs
- 282 lbs
- 283 lbs
- 284 lbs
- 285 lbs
- 286 lbs
- 287 lbs
- 288 lbs
- 289 lbs
- 290 lbs
- 291 lbs
- 292 lbs
- 293 lbs
- 294 lbs
- 295 lbs
- 296 lbs
- 297 lbs
- 298 lbs
- 299 lbs
- 300 lbs
- More than 300 lbs

10. How tall are you (in feet and inches)?
    - Less than 4 feet 0 inches
    - 4 feet 0 inches
    - 4 feet 1 inches
    - 4 feet 2 inches
    - 4 feet 3 inches
    - 4 feet 4 inches

- 4 feet 5 inches
- 4 feet 6 inches
- 4 feet 7 inches
- 4 feet 8 inches
- 4 feet 9 inches
- 4 feet 10 inches
- 4 feet 11 inches
- 5 feet 0 inches
- 5 feet 1 inches
- 5 feet 2 inches
- 5 feet 3 inches
- 5 feet 4 inches
- 5 feet 5 inches
- 5 feet 6 inches
- 5 feet 7 inches
- 5 feet 8 inches
- 5 feet 9 inches
- 5 feet 10 inches
- 6 feet 0 inches
- 6 feet 1 inches
- 6 feet 2 inches
- 6 feet 3 inches
- 6 feet 4 inches
- 6 feet 5 inches
- 6 feet 6 inches
- 6 feet 7 inches
- 6 feet 8 inches
- More than 6 feet 8 inches

11. What is your racial and ethnic background?
    - White
    - Black
    - Asian
    - Hispanic or Latino
    - Other
    - Rather not say

12. What is your marital status?
    - Single
    - Divorced
    - Separated
    - Widowed
    - Married/in a domestic partnership
    - Not married/in a domestic partnership but living with significant other
    - In a relationship, but not living in the same home as your significant other

13. What is the highest degree or level of school you have completed?
    - Less than a high school diploma
    - High school degree or equivalent
    - Bachelor’s degree (e.g. BA, BS)
    - Master’s degree (e.g. MA, MS, Med)

- Doctorate (e.g. PhD, MD, EdD)
- Other

14. Are you pregnant at the moment?
    - Yes
    - No

NEXT PAGE

In this section, we will ask you about your usual eating patterns during your pregnancy (current or in most recent pregnancy).

30% Completed [progress bar]

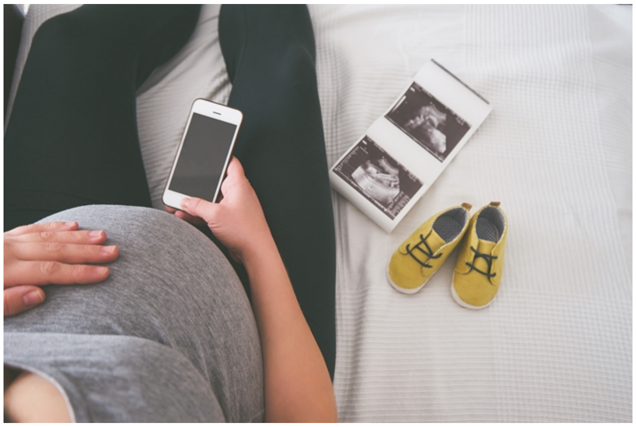

15. Compared to before you were pregnant, how would you rate your changes to the following eating behaviors when pregnant? (If you are currently not pregnant, please answer for when you were pregnant)

	**A lot less**	**A little less**	**About the same**	**A little more**	**A lot more**	**I don't know**
Taking a daily vitamin						
Eating healthier foods						
The amount of food that you eat						
The size of your meals						
How often you snack						
The amount of sugar that you eat						
The amount of fat that you eat						
The amount of salty foods that you eat						
The amount of fried foods or fast foods you eat						
The amount of fruit that you eat						
The amount of vegetables that you eat						
The amount of fiber that you eat						
The time between your last meal or snack and the time you go to bed						

How often you eat breakfast						
How often you wake up in the middle of the night to eat or drink something (other than water)						
How often you drink sugar-sweetened beverages (soda, juice, sweetened tea, smoothies etc.)						
How often you count calories to eat less						

NEXT PAGE

45% Completed [progress bar]

16. When pregnant, how many meals do you typically eat per day? Meals refer to food eaten that contains the calories and nutrients you need to keep you full, such as breakfast, lunch, and dinner.
    - 0
    - 1
    - 2
    - 3
    - 4
    - 5 or more

17. When pregnant, how many times do you snack per day? Snacks are foods or drinks (other than water) that you eat or drink between meals.
    - 0
    - 1
    - 2
    - 3
    - 4
    - 5 or more

18. When pregnant, after you wake up in the morning, how long is it before you eat your first meal or snack of the day?
    - Pretty much as soon as I wake up
    - After about 30 minutes
    - After about 1 hour
    - After about 1½ hours
    - After about 2 hours
    - After about 3 hours
    - After about 4 or more hours

19. When pregnant, do you typically drink anything other than water, unsweetened tea, or black coffee before your first meal or snack of the day? For example, juice, soda, tea/coffee with cream or sugar etc.
    - Yes
    - No

20. When pregnant, when do you usually eat your last meal before going to bed?
    - Right before bed (within 30 minutes)
    - 1 hour before bed

- 1½ before bed
- 2 hours before bed
- 3 hours before bed
- 4 or more hours before bed

21. When pregnant, do you usually consume any extra snacks or beverages (other than water or calorie free beverages) after your last meal of the day?
    - Yes
    - No

22. **If "Yes" is selected to question 21:** How many minutes before bed do you consume this snack or beverage?
    - Right before bed (within 30 minutes)
    - 1 hour before bed
    - 1½ hours before bed
    - 2 hours before bed

23. When pregnant, do you typically wake up in the middle of the night for a snack or drink (other than water)?
    - Yes
    - No

24. When pregnant, do you wake up hungry in the mornings?
    - Yes
    - No

25. When pregnant, do you wake up hungry in the middle of the night?
    - Yes
    - No

26. When pregnant, have you followed any of these diets? If not, would you be willing to work with a dietician to follow one of these eating patterns during the second or third trimester of pregnancy?

	**I HAVE TRIED this diet during pregnancy**	**I HAVE TRIED this diet during pregnancy and did NOT like it**	**I have not tried this diet and WOULD NOT be willing to try during my pregnancy**	**I HAVE NOT TRIED this diet but would be willing to try it during my pregnancy**
Restricting eating to only a certain number of hours per day (also called Time-Restricted Eating)				
Eating normally on 5 days per week and restricting eating on 2 days per week (also called the 5:2 diet)				
Eating every other day (also called the Alternate Day Fasting diet)				

Any other type of fasting not described above				
Eating a Paleo, Ketogenic, Atkins, or similar low carb diet				
Eating Mediterranean or DASH diet				
Eating a Whole-30 diet				
Eating a Vegetarian diet				
Eating a Vegan diet				
Calorie counting				

NEXT PAGE

Researchers at the Pennington Biomedical Research Center (Baton Rouge, Louisiana) have shown that eating all your calories per day in a window of 6-8 hours and fasting (not eating) for 16-18 hours per day decreased appetite and had beneficial changes on health. Fasting excludes all foods and drinks except for water and non-caloric drinks.

In this section, we will ask you how you would feel about following an eating plan that asks you to fast for a certain amount of time during your second or third trimester of pregnancy.

60% Completed [progress bar]

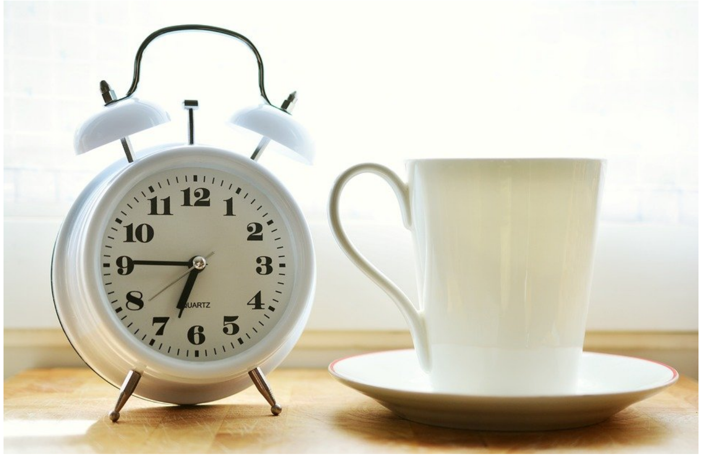

27. During pregnancy, how many hours would you be willing to fast (or not eat) between meals and snacks? For example, if you are your dinner at 7:00 PM and did not eat again until 9:00 AM, this would be a 14 hour fast.
    - Less than 5 hours
    - I could fast for 6 hours
    - I could fast for 7 hours
    - I could fast for 8 hours
    - I could fast for 9 hours
    - I could fast for 10 hours
    - I could fast for 11 hours

- I could fast for 12 hours
- I could fast for 13 hors
- I could fast for 14 hours
- I could fast for 15 hours
- I could fast for 16 hours
- I could fast for 17 hours
- I could fast for more than 18 hours

28. During pregnancy, would you prefer fasting in the morning (skip breakfast and start eating for the day around lunch time) or fasting in the evening before bed (start eating in the morning and end eating later afternoon/early evening)?
    - Fast in the morning (skip breakfast and start eating around lunch time
    - Fast in the evening before bed (start eating in the morning and end eating late afternoon/early evening)

29. What concerns would you have about eating all of your meals over 8 hours, starting in the morning and fasting for 16 hours overnight, during your second or third trimester of pregnancy?

	**Not at all concerned**	**Not very concerned**	**Somewhat concerned**	**Very concerned**	**I don't know**
I would be too hungry					
I would get too nauseous					
I would not eat enough food					
It would be hard to eat a balanced diet					
It would not be safe for my health					
It would not be safe for my baby's health					
It would not work with my busy schedule					
It would be hard to follow if my family doesn't join me					

30. If you could work with a dietician/nutritionist during your pregnancy (free of charge) to follow a new eating plan that involved eating for a shorter time period, like 8 hours per day, and fasting for about 16 hours overnight, would you find these things be helpful?

	**Not at all helpful**	**Not very helpful**	**Somewhat helpful**	**Very helpful**	**I don't know**
Being able to choose my own eating time window					
Following the time-restricted eating plan for 5 days out of the week only					
Being able to begin eating in the morning and stopping around 5pm so that I would fast overnight					

Being able to video chat with my dietician/nutritionist while I eat my dinner, since it will be outside the range of time that my family eats dinner					
Being able to begin eating at lunch so I could stop eating around 8pm (this means fasting less at night but fasting until lunch, or around 12pm)					
Using an app to log my eating times					
Getting feedback from my dietician/nutritionist on my app logs so I can make sure what I am eating is healthy					
Checking in with my doctor more often to make sure my baby is healthy					

NEXT PAGE

In this section, we will ask you about your exercise habits during your pregnancy

75% Completed [progress bar]

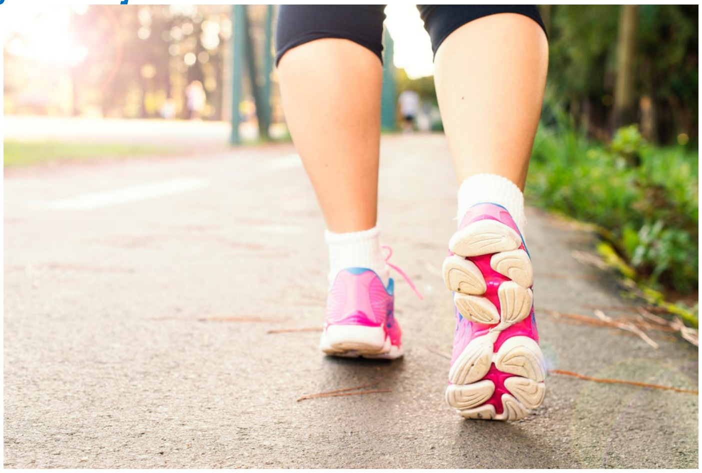

31. When pregnant, do you perform the following exercises?

	Yes	No
Cardio - "Cardio" exercises increase your heart rate and breathing rate and make it difficult to hold conversation while working out. Examples of "cardio" exercises include: walking briskly, running or jogging, using the elliptical machine, dance, or Zumba classes.		
Yoga - includes prenatal yoga		
Weight lifting or resistance training		
Pilates		
HIIT Classes (High Intensity Interval Training)		

32. **If "Yes" is selected to "Cardio":** How many days per week do you perform cardio exercises during pregnancy?

- 1 day per week
- 2 days per week
- 3 days per week
- 4 days per week
- 5 days per week
- 6 days per week
- 7 days per week

NEXT PAGE

Pregnant women are recommended to exercise for at least 150 minutes per week, spread throughout the week (for example, 30 minutes a day for 5 days).

90% Completed [progress bar]

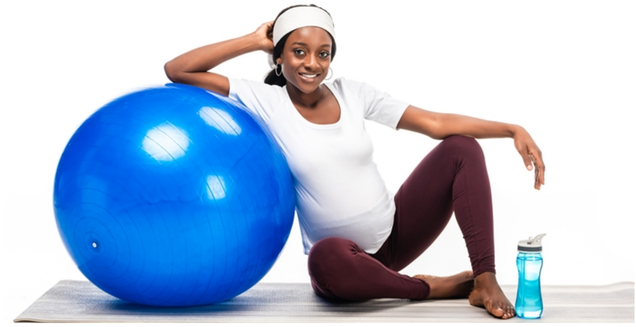

33. How many days per week would you be willing to exercise to meet 150 minutes per week during your second or third trimester of pregnancy?

	NOT AT ALL WILLING	NOT VERY WILLING	SOMEWHAT WILLING	VERY WILLING	I DON'T KNOW
1 day per week					
2 days per week					
3 days per week					
4 days per week					
5 days per week					
6 days per week					
7 days per week					

34. What concerns would you have about performing exercise for 150 minutes per week during your second or third trimester of pregnancy?

	Not at all concerned	Not very concerned	Somewhat concerned	Very concerned	I don't know
I would not enjoy the exercise					

The exercise would be too hard					
I would be too tired					
I would be too uncomfortable					
It would not be safe for my health					
It would not be safe for my baby's health					
It would not work with my busy schedule					
I would not have childcare for my other children					
It would take away time from my social life					

35. If you could work with a professional exercise specialist (free of charge) in a secure, private, and sanitized environment to follow a workout plan during your second or third trimester of pregnancy, would you find these things helpful?

	**Not at all helpful**	**Not very helpful**	**Somewhat helpful**	**Very helpful**	**I don't know**
Being able to choose the time of day for my workouts					
Being able to do some of the workout sessions at home					
Being able to do some of the workout sessions at home while video chatting with my exercise specialist					
Working out on weekdays only					
Working out on weekends only					
Having the workout sessions catered to my personal fitness level					
Checking in with my doctor more often to make sure my baby is healthy					
Providing childcare for my other children while I workout					

NEXT PAGE

95% Completed [progress bar]

36. Would you be willing to answer a few more questions on eating patterns during pregnant? (~5 minutes)
    - ○ Yes
    - ○ No

If "No" is selected to question 35, the survey displays the following verbiage:

End the survey?

You have selected an option that triggers this survey to end right now. To save your responses and end the survey, click the button below to do so. If you have selected the wrong option by accident and do not wish to leave the survey, you may click the other button below to continue, which will also remove the value of the option you just selected to allow you to enter it again and continue the survey.

Continue survey and undo last response **End the survey now**

Participants are directed to a final page with one additional question and compensation text box.

37. Do you have any more thoughts or concerns on exercise or diet programs during pregnancy? (OPTIONAL)

THANK YOU FOR COMPLETING OUR SURVEY!

If you would like to be considered for one of our ten $50 gift cards, please enter your email address here. Please note that if you are selected to receive one of the gift cards, more information will be needed during follow-up.

____

If "Yes" is selected to question 36, the following questions appear on the same page.

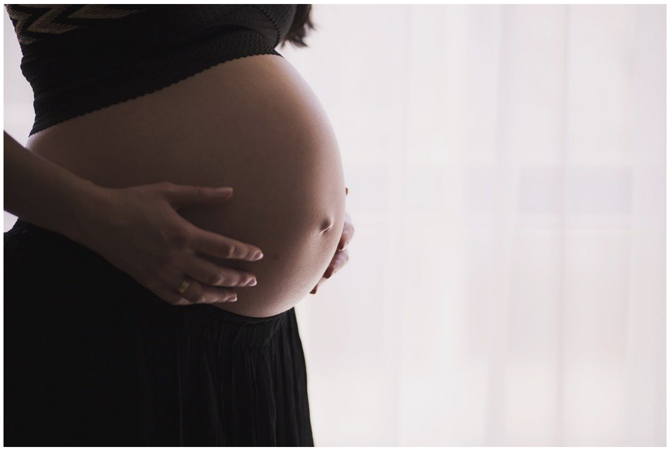

38. How safe would you rate following these diets during your second and third trimester of pregnancy?

	Not at all safe	Not very safe	Somewhat safe	Very safe	I don't know
Some variation of fasting or Time-Restricted Eating					
Calorie counting to eat less					
Eating low fat foods					
Eating low carb foods					
Eating low sugar foods or eliminating sugar					
Eating a Paleo, Ketogenic, Atkins, or similar diet					
Eating a Mediterranean diet					
Whole-30					
Eating a Vegetarian Diet					
Eating a Vegan Diet					

39. What concerns would you have about following these eating plans during your second or third trimester of pregnancy?

	Concern for Vegetarian/Plant-based diet	Concern for Paleo, Ketogenic, Atkins, or similar low-carb diet	Concern for BOTH of these diets	NOT a concern for either of these diets
I would not enjoy the food				
I would be too hungry				
I would get nauseous				
I would not eat enough food				
It would be hard to eat a balanced diet				
I would not get enough protein				
I would not get enough fat				
It would not be safe for my health				
It would not be safe for my baby's health				
It would not work with my busy schedule				
It would be expensive				
It would be hard to follow if my family doesn't join me				

40. Select if these things would make following each diet MORE HELPFUL.

	Helpful for Vegetarian/Plant-based diet	Helpful for Paleo, Ketogenic, Atkins, or	Helpful for BOTH of these diets	NOT helpful for either

		similar low-carb diet		of these diets
Being provided protein shakes or bars to supplement my own diet				
Being provided all of my meals and snacks				
Following this diet on only 5 days of the week				
Meeting with a dietician/nutritionist periodically				
Checking in with my doctor more often to make sure my baby is healthy				

41. Do you have any more thoughts or concerns on exercise or diet programs during pregnancy? (OPTIONAL)

THANK YOU FOR COMPLETING OUR SURVEY!

If you would like to be considered for one of our ten $50 gift cards, please enter your email address here. Please note that if you are selected to receive one of the gift cards, more information will be needed during follow-up.

Thank you.
