# Supplementary material for: Eating Behaviors and Dietary Patterns of Women during Pregnancy: Optimizing the Universal ‘Teachable Moment’
Source: Nutrients. 2021 Sep 21;13(9):3298. doi: 10.3390/nu13093298 (PMC8471126; doi:10.3390/nu13093298)
Supplement: Supplementary file 1 [file nutrients-13-03298-s001.zip › Supplementary Table 1.pdf]

Table S1. Significant relationships for women with obesity compared to normal-weight women.

**Eating behaviors**[a]	**Less vs. Maintain**			**Less vs. More**			**More vs. Maintain**		
	**OR**	**CI**	**P-value**	**OR**	**CI**	**P-value**	**OR**	**CI**	**P-value**
Healthfulness of food	0.81	0.39-1.69	0.58	1.73	0.87-3.44	0.12	2.07	1.31-3.29	**p=0.002**
Amount of food	0.29	0.12-0.63	**p=0.002**[a]	0.23	0.11-0.47	**p<0.0001**[b]	0.75	0.46-1.22	0.25
Size of meals	0.51	0.30-0.86	**P=0.012**	0.56	0.33-0.96	**p=0.036**	1.10	0.67-1.81	0.71
Snacking frequency	0.34	0.14-0.84	**p=0.019**	0.30	0.12-0.67	**p=0.003**	0.84	0.50-1.39	0.49
Amount of fat	0.45	0.24-0.85	**p=0.014**	0.35	0.16-0.75	**p=0.007**[c]	0.75	0.45-1.27	0.28
Amount of fried or fast foods	0.55	0.35-0.88	**p=0.012**	0.79	0.42-1.47	0.45	1.30	0.71-2.38	0.39
Amount of fruit	0.34	0.12-0.93	**p=0.036**	0.31	0.12-0.79	**p=0.015**	0.81	0.52-1.27	0.36

[a]Partly determined by Caucasian race (3.40 [CI 1.45-8.02], p=0.005).

[b]Partly determined by higher education levels (0.46 [CI 0.22-0.95], p=0.035).

[c]Partly determined by Caucasian race (2.65 [CI 1.39-5.0], p=0.003).
